# Ultrasound-guided caudal epidural steroid injection in chronic radicular low back pain: short-term electrophysiologic benefits

**DOI:** 10.1259/bjro.20190006

**Published:** 2020-01-13

**Authors:** Maha Emad Ibrahim, Magdy Ahmed Awadalla, Aziza Sayed Omar, Mohammad al-Shatouri

**Affiliations:** 1 Lecturer of Physical Medicine, Rheumatology and Rehabilitation, Faculty of Medicine, Suez Canal University. PhD in Physical Medicine, Rheumatology and Rehabilitation, Ismailia, Egypt; 2 Professor of Physical Medicine, Rheumatology and Rehabilitation, Faculty of Medicine, Suez Canal University. PD in Physical Medicine, Rheumatology and Rehabilitation, Ismailia, Egypt; 3 Associate professor of Radiology, Faculty of Medicine, Suez Canal University, Ismailia, Egypt

## Abstract

**Objective:**

To assess the short-term efficacy of ultrasound-guided caudal epidural steroid injections (ESIs) in improving pain, and nerve function as measured by electrophysiological testing in chronic radicular low back pain.

**Methods:**

Patients diagnosed with chronic radicular low back pain were randomized into one of two groups. The injection group (*n* = 20) underwent a single ultrasound-guided Caudal ESI of 1 ml of 40 mg ml^−1^ Triamcinolone Acetonide (Kenacort-A), with local anesthetic. The control group (*n* = 20) underwent a 12-session physiotherapy program. Both groups were evaluated before and 2 weeks after the intervention using visual analog scale for pain and electrophysiological testing comprising peroneal and tibial terminal motor latencies and F-response latencies and chronodispersion.

**Results:**

Both groups showed significant pain reduction on the visual analog scale after the intervention. The injection group showed a significant reduction in F wave chronodispersion post-treatment (<0.01). In the control group, there were no significant differences in F wave parameters pre- and post-treatment (*p* > 0.05).

**Conclusion:**

Caudal ESIs were shown to provide short-term improvement of nerve function as evident by improvement in the electrophysiological parameters sensitive to radiculopathy. It was found to be superior to standard physical therapy in this regard.

**Advances in knowledge:**

This work shows a novel electrophysiologic evidence of the short-term efficacy ultrasound-guided caudal ESI.

## Introduction

Chronic lumbar radiculopathy is characterized by back and leg pain associated with sensory, motor or reflex deficits in a distribution of nerve root lasting for more than 12 weeks^1,2^.^2^ The estimated prevalence of lumbar radiculopathy has been described as 9.8 per 1000 cases,^3^ 5.1% in males and 3.7% in women.^4^ In addition to its high prevalence, it is a leading factor in lost productivity, disability, and medical expenditures.^5^

Epidural injections are among the most commonly performed interventions for managing radicular pain.^6,7^ The caudal epidural procedure is one of the three approaches available to access the lumbar epidural space in addition to interlaminar and transforaminal approaches. It has the advantage of being target specific for lower levels, with ability of reaching the ventrolateral epidural space, and it can be safely performed in cases of failed back syndrome.^8,9^ Another advantage is its lower risk of dural injury.^10^

It is important to identify if nerve root injury is present and which roots are affected. Studies that establish physiological abnormalities are important and can act as functional measures that reflect the extent of root affection, combined with the clinical and radiologic data. Electrophysiological testing of the late responses (in which the latency exceeds that of the M-wave) can be helpful in this regard. These are superior in their ability to study the proximal nerve segments where the radicular pathology lies.^11^

F waves represents the late response produced by supramaximal stimulation resulting in antidromic activation of the motor neurons. The most frequently used parameter in the evaluation of F raves is the Latency.^12^ Although the minimum latency is reported most commonly,^13^ mean F wave latencies may dilute the errors of measurement and produce more reliable results.^14^

The F waves is less sensitive than needle electromyography in radiculopathies^15,16^; the only parameter used was the minimal latency. Normal latencies may occur even if an individual root is injured due to multiple root innervations of the muscles. Unlike minimal F wave latencies that can be normal due to presence of a small number of normally conducting axons, F wave chronodispersion can provide a measure of the range of conductions in an injured nerve root. F wave parameters combined with minimal F wave latencies can provide a sensitivity for radiculopathy similar to the sensitivity of needle electromyography.^11,17–19^ Furthermore, the needle study is much less sensitive to the process of reinnervation than it is to recent denervation, therefore not very useful as a follow-up study. Based on prolonged latencies and chronodispersion; The F waves showed a sensitivity of 50–80% in previous reports.^20^

Most studies evaluating the effectiveness of epidural steroid injection (ESIs) utilizes subjective measures, including visual analog scale (VAS) and functional assessments. Here, we aimed to evaluate the electrophysiological outcomes of ESIs, using the F-wave as a tool reflective of root pathology for evaluating the nerve function.

## Methods and materials

This study was a randomized controlled clinical trial on patients diagnosed with chronic radicular low back pain (LBP) attending the Physical Medicine, Rheumatology and Rehabilitation Department. The study was approved by the local institutional review board.

40 consecutive subjects presenting with radicular LBP were enrolled. The participants were fulfilling the study criteria and provided written informed consents. Radicular LBP was defined as pain that radiates into the lower extremity directly along the course of a spinal nerve root.^5^

All of the patients were subjected to a detailed medical history, physical examination and lumbosacral MRI study. Subjects were included if they were diagnosed clinically with radicular LBP by satisfying the following criteria combined: (1) a history showing LBP radiating in the distribution of L4, L5, or S1 dermatomes; (2) MRI showing significant nerve impingement of the L4, L5, and/or S1 nerve roots (L3/L4, L4/L5 and L5/S1 root lesions); (3) physical examination showing radicular irritation or abnormalities in the sensory, motor, and/or reflex of the L4, L5, or S1 nerve root distribution.

Patients with cauda equina symptoms, peripheral and entrapment neuropathies, uncontrolled psychiatric illnesses, inability to achieve proper positioning or with history of adverse reaction to local anesthetic and history of gastrointestinal bleeding were excluded from the study.

## Study protocol

### Assessment

Patients enrolled were randomized into one of two groups; the study group**,** which included 20 patients diagnosed with radicular LBP who received a single ultrasound-guided caudal ESI, and the control group which included 20 patients diagnosed with radicular LBP who received 12 sessions of physical therapy.

Each patient had a history and physical examination, including motor and sensory evaluation, muscle examination, straight leg raising test, muscle stretch reflexes, and VAS for radicular pain. The patients then were subjected to routine motor nerve conduction studies, F response of the common peroneal and posterior tibial nerves of the affected limb as follows:

Electrophysiological study was carried out in a quiet room. The device used was a NIHON KOHDEN Neuropack MEB-7102 mobile unit with a two-channel evoked potential/electromyography measuring system (Nihon Kohden Corporation, Tokyo, Japan). The test was performed with the patient comfortably seated in a semi-sitting position during the whole test period lasting 15–30 min, with skin temperature maintained around 32–34°C.

#### Technique

1- Compound motor action potentials (CMAPs) of both posterior tibial and peroneal nerves were recorded as follows:

The active (G1) electrode was placed over the muscle belly (Abductor Hallucis and Extensor digitorum Brevis), and the reference (G2) over the tendon. A ground electrode was placed between the recording and stimulating electrodes. The sensitivity was set at 5–10 mV per division. Standard bipolar surface stimulation was used for motor nerve conduction studies where the cathodal pole is oriented proximal to the anode. A supramaximal stimulation was attempted, terminal motor latency, amplitude and NCV were recorded.^21^

#### F- wave were recorded as follows

With the same setting of the CMAP**,** the cathode was positioned at the exact same site utilized for distal stimulation at the ankle. The anode was then rotated 180° from this position for obtaining the distal motor latency. Stimulus intensity was adjusted to be supramaximal (about 20–30% supramaximal). Stimuli are delivered at a frequency of 0.5 Hz (every 2 s) or less. 30–40 consecutive stimuli were administered.^22^

Analysis of the response was done using minimum F-wave latency which is defined as the shortest latency to onset of the initial deflection (either negative or positive) of all recorded F waves, maximum F-wave latency defined as the longest latency to onset of the initial deflection, mean F-wave latency which is the average latency of all recorded F waves and chronodispersion: the difference in the shortest and longest of the F-wave onset latencies.

Normal values for mean latencies were calculated according to height, chronodispersion upper limit for normal was 9.5 ms for the peroneal and 9.3 ms for the tibial nerve.^23^

### Interventions

#### Study group

**Figure 1.**
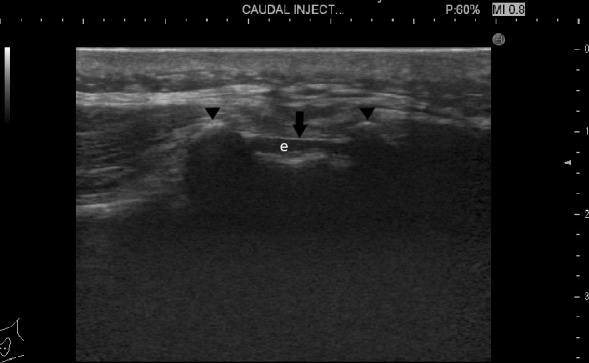
An ultrasound image of the sacral hiatus transverse view showing the sacral cornue (arrowheads), the sacrococcygeal membrane (arrow) and the epidural space (e).

**Figure 2.**
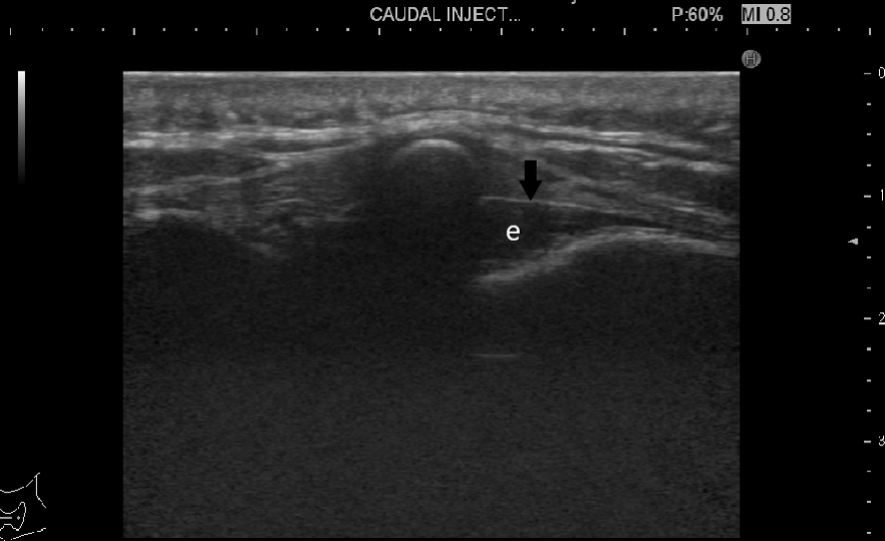
An ultrasound image of the sacral hiatus sagittal view showing the sacrococcygeal membrane (arrow) and the hypo-echoic epidural space (e).

Patients in this group received a single caudal ESI. None of the patients received a second injection during the study period in order not to add confounding factors. The interventional procedures were done by a single radiologist (author MA) with 7 years experience in ultrasound-guided spinal interventional radiology. The patient lied in the prone position. A Toshiba Nemio XG ultrasound machine with a linear probe (8–13 MHz) was used. The sacral hiatus was identified by ultrasound using the following landmarks:

Using a transverse probe orientation, the sacral cornua are seen as two echogenic structures forming a (U) shape. Two echogenic band-like structures can be identified between them, the sacrococcygeal ligament superficially and the bony surface of the sacrum deep to it. The sacral hiatus is seen as echogenic region between these two band-like surface (Figure 1). The probe was then rotated 90° oriented at the midline sagittal view to show the epidural space (Figure 2).

**Figure 3.**
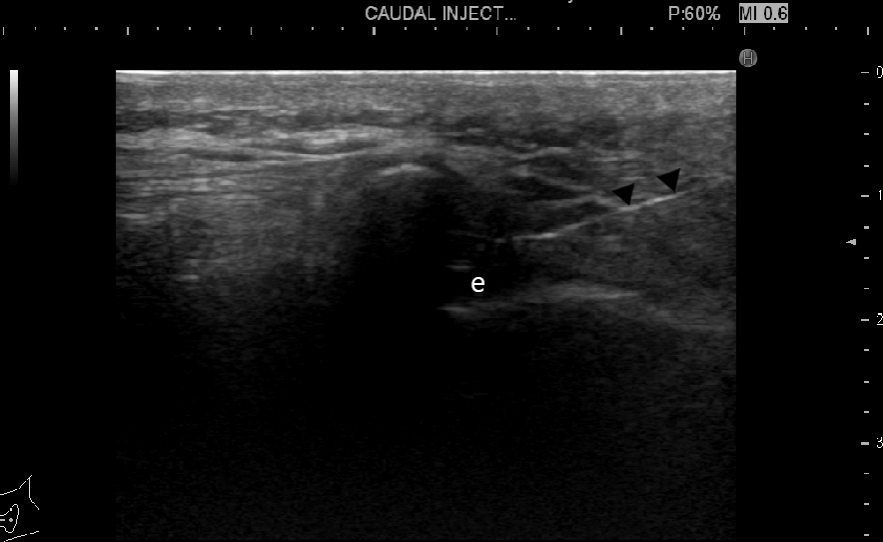
An ultrasound image of the sacral hiatus sagittal view showing the needle (arrowheads) in place with its tip at the epidural space (e).

**Figure 4.**
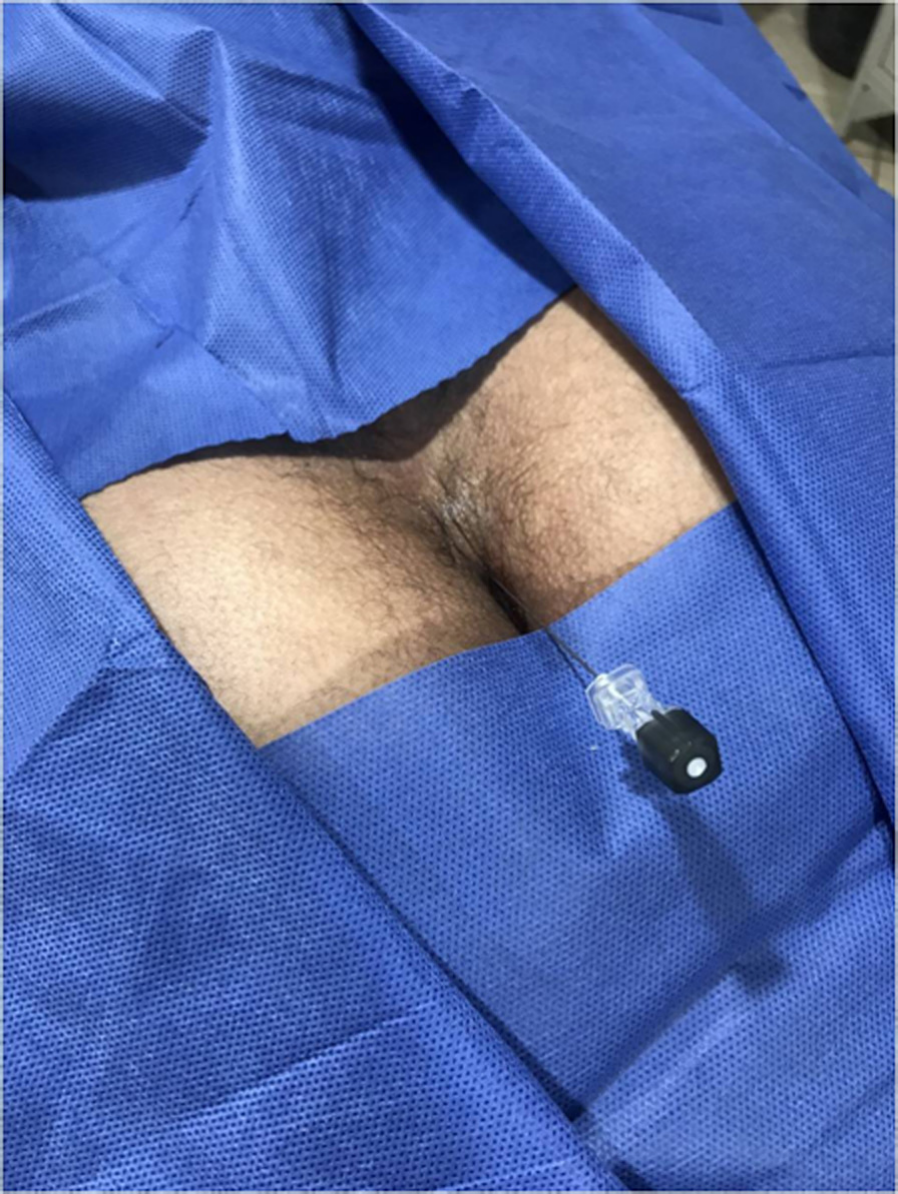
A photo showing the spinal needle entering the caudal region with the patient prone and the needle directed caudocranial.

Power Doppler can be applied to check for any vessels at the expected path (6.7 MHz, pulse repetition frequency of 600 Hz). The skin was cleansed twice using povidone-iodine followed by a 70% alcohol, then covered with a sterile drape with an opening placed over the injection field. The ultrasound probe was covered with sterile plastic. A 25G needle was first used for skin and subcutaneous anesthesia using a 3 ml of 2% lodocaine. A 22-gauge spinal needle was then advanced into the sacral hiatus under real-time ultrasound, using the longitudinal probe orientation. Passage of the needle through the ligament was perceived as resistance followed by a release when the needle enters the canal epidural fat. The ultrasound can confirm the needle tip portion (Figures 3 and 4). Gentle aspiration is done to ensure the absence of blood or cerebrospinal fluid. Once proper needle position was established, 1 ml of 40 mg ml^−1^ Triamcinolone Acetonide (Kenacort-A) mixed with 3 ml lidocaine 2% and 6 ml saline (Sodium Chloride 0.9%) was injected for proper distribution of the drugs. The needle was then left in place for 1 min with its hub closed to allow time for proper drug destruction and ensure absence of backflow of drugs to the subcutaneous tissues if the needle was immediately removed.

#### Control group

Patients in this group received 12 sessions of physical therapy carried out 3 days/week for 4 weeks, including superficial heat, therapeutic interrupted ultrasound to paraspinal muscles, transcutaneous electrical nerve stimulation, therapeutic exercise and manual massage.

Both groups were allowed to take simple analgesics (acetaminophen 500 mg tablets thrice-daily after meals) throughout the study duration.

### Follow up

All patients were re-evaluated 2 weeks after the procedures by clinical examination, VAS for pain and electrophysiological evaluation.

#### Statistical analysis and data management:

Data were coded, entered using Microsoft Excel software, then imported into Statistical Package for the Social Sciences (SPSS v. 18.0) for analysis. According to the type of data; χ^2^, McNemar test, paired *t*-test with least significance difference were used to test differences for significance between pre- and post-assessments. *p-*Value was set at <0.05 for significant results.

## Results

The mean age for cases was 52.85 ± 9.72 years, while that for controls was 45.75 ± 11.85 years, 35% of cases and 30% of controls had symptoms lasting more than 24 months, and 85% of both cases and controls were females. 60% of cases and 50% of controls had root compression on multiple levels. Among cases with single root compression 6 (30%) had L5 lesion and 2 (10%) had S1 lesion. Among controls 3 (15%) had L4 lesion, 3 (15%) had L5 lesion, and 4 (20%) had S1 lesions. There was no statistically significant difference between cases and controls regarding age, duration of symptoms, weight, height, concomitant illnesses or level of root lesions. The interventional procedures were all successful with no any technical failure among the studied group.

Mean VAS before the intervention among patients in the intervention group was 9.16 ± 0.8. While the mean VAS post-intervention among the same patients was 5.13 ± 1.85. In the control group, mean VAS pre-intervention was 8.15 ± 1.34, and after the intervention became 5.15 ± 1.46. Both groups show statistically significant differences between pre- and post-intervention measurements. However, when comparing the improvement in VAS after the intervention (difference in difference), and the number of patients achieving significant pain reduction (as defined by a reduction of 2 or more points on the scale) between both groups the results were not statistically significant (*p* > 0.05) (Table 1).

**Table 1.** Mean VAS for pain pre- and post-intervention

Patients	Pre-intervention Mean ± SD	Post-intervention Mean ± SD	Percentage of improvement	*p*-value
Cases (*n* = 20)	9.16 ± 0.9	5.13 ± 1.85	40.3%	<0.01**
Controls (*n* = 20)	8.15 ± 1.34	5.15 ± 1.46	30%	<0.01**

VAS, visual analog scale.

a Significant *p*-value <0.01, paired sample *t*-test.

Regarding the electrophysiological parameters of the common peroneal nerve measured pre-intervention, 65% of cases, and 75% of controls had abnormal F-wave mean latency (either delayed or absent F wave), While 95% of cases and 85% of controls had abnormal chronodispersion of the common peroneal nerve. There were no statistically significant differences between cases and controls regarding all measured common peroneal nerve electrophysiological parameters before the intervention (Table 1).

As for the posterior tibial nerve electrophysiological parameters pre-intervention, 70% of cases and 60% of controls had a delayed/absent F wave chronodispersion pre intervention. There were no significant differences between cases and controls regarding all measured posterior tibial nerve parameters before the intervention (Table 2).

**Table 2.** Electrophysiological parameters of the common peroneal and posterior tibial nerves among cases and controls pre-intervention

Common peroneal nerve			
Electrophysiological parameters	Cases (*n* = 20) Mean ± SD	Controls (*n* = 20) Mean ± SD	*p-*value
Distal latency	4.85 ± 1.5	4.08 ± 0.94	0.06
Amplitude	3.77 ± 2.5	4.82 ± 2.5	0.20
NCV	48.68 ± 4.36	48.39 ± 6.6	0.87
F-wave mean latency			
Normal	7 (35%)	5 (25%)	0.77
Delayed	4 (20%)	4 (20%)	
Absent	9 (45%)	11 (55%)	
Mean ± SD	46.78 ± 2.99	46.69 ± 4.3	0.95
F-wave minimum latency	40.81 ± 4.76	41.27 ± 6.41	0.86
F-wave maximum latency	53.75 ± 3.42	51.91 ± 2.97	0.92
F-wave chronodispersion			
Normal	1 (5%)	3 (15%)	0.33
Delayed	10 (50%)	6 (30%)	
Absent	9 (45%)	11 (55%)	
Mean ± SD	12.95 ± 4.2	12.64 ± 2.97	0.89
**Posterior tibial nerve**			
Distal latency	5.16 ± 1.16	4.93 ± 1.23	0.56
Amplitude	9.55 ± 4.36	13 ± 8.33	0.11
NCV	49.34 ± 6.11	44.07 ± 11.9	0.09
F-wave mean latency			
Normal	7 (35%)	11 (55%)	0.43
Delayed	12 (60%)	8 (40%)	
Absent	1 (5%)	1 (5%)	
Mean ± SD	48.88 ± 4.76	46.28 ± 4.34	0.09
F-wave minimum latency	44.71 ± 4.41	42.14 ± 6.7	0.17
F-wave maximum latency	53.8 ± 3.1	50.35 ± 3.9	0.06
F-wave chronodispersion			
Normal	6 (30%)	8 (40%)	0.80
Delayed	13 (65%)	11 (55%)	
Absent	1 (5%)	1 (5%)	
Mean ± SD	9.08 ± 3.98	9.36 ± 6.33	0.64

NCV, nerve conduction velocity; SD, standard deviation.

a Significant *p*-value <0.05, *t*-test or χ^2^ test, as appropriate.

In comparing common peroneal electrophysiological parameters pre- and post-intervention measurements among cases, there is a significant reduction of the mean F-wave chronodispersion. The mean F wave also decreased, but the difference was not statistically significant. The number of patients having normal chronodispersion increased significantly. Comparing the posterior tibial nerve parameters among cases also showed a significant reduction of the mean F-wave chronodispersion after the intervention, and the number of patients having normal chronodispersion increased significantly from 30 to 95% (*p* < 0.01) (Table 3).

**Table 3.** Electrophysiological parameters of common peroneal and posterior tibial nerves among cases pre- and post-intervention (*n* = 20)

Common peroneal nerve			
Electrophysiological parameters	Pre-intervention Mean ± SD	Post-intervention Mean ± SD	*p-*value
Distal latency	4.85 ± 1.5	4.27 ± 1.5	0.53
Amplitude	3.77 ± 2.5	4.03 ± 2.17	0.72
NCV	48.68 ± 4.36	46.26 ± 6.4	0.30
F-wave mean latency			
Normal	7 (35%)	7 (35%)	0.06
Delayed	4 (20%)	10 (50%)	
Absent	9 (45%)	3 (15%)	
Mean ± SD	46.78 ± 2.99	44.23 ± 4.33	0.17
F-wave minimum latency	40.81 ± 4.76	40.14 ± 6.62	0.81
F-wave maximum latency	53.75 ± 3.42	48.91 ± 6.60	0.04*
F-wave chronodispersion			
Normal	1 (5%)	17 (85%)	<0.01**
Delayed	10 (50%)	0 (0%)	
Absent	9 (45%)	3 (15%)	
Mean ± SD	12.95 ± 4.2	8.87 ± 1.25	<0.01**
**Posterior tibial nerve**			
Distal latency	5.16 ± 1.16	4.62 ± 0.96	0.06
Amplitude	9.55 ± 4.36	9.75 ± 5.96	0.89
NCV	49.34 ± 6.11	44.48 ± 4.59	<0.01**
F-wave mean latency			
Normal	7 (35%)	10 (50%)	0.43
Delayed	12 (60%)	10 (50%)	
Absent	1 (5%)	0 (0%)	
Mean ± SD	48.88 ± 4.76	47.15 ± 3.99	0.25
F-wave minimum latency	44.71 ± 4.41	45.39 ± 3.25	0.59
F-wave maximum latency	53.8 ± 3.1	51.93 ± 2.92	0.052
F-wave chronodispersion			
Normal	6 (30%)	19 (95%)	<0.01**
Delayed	13 (65%)	1 (5%)	
Absent	1 (5%)	0 (0%)	
Mean ± SD	9.08 ± 3.98	6.35 ± 2.09	0.01*

NCV, nerve conduction velocity; SD, standard deviation.

a Significant *p*-value <0.05, ** significant *p*-value <0.01, paired *t*-test or Mc Nemar test, as appropriate.

Comparing measurements of both peroneal and tibial nerves between the pre- and post-intervention measurements among controls, showed that none of the measured parameters showed a significant improvement after the intervention (Table 4).

**Table 4.** Electrophysiological parameters of common peroneal and posterior tibial nerves among controls pre- and post-intervention (*n* = 20)

Electrophysiological parameters	Pre-intervention Mean ± SD	Post-intervention Mean ± SD	*p-*value
**Common peroneal nerve**			
Distal latency	4.08 ± 0.94	4.53 ± 1.18	0.12
Amplitude	4.82 ± 2.5	4.46 ± 2.34	0.60
NCV	48.39 ± 6.6	47.62 ± 5.7	0.66
F-wave mean latency			
Normal	5 (25%)	7 (35%)	0.76
Delayed	4 (20%)	4 (20%)	
Absent	11 (55%)	9 (45%)	
Mean ± SD	46.69 ± 4.3	45.83 ± 5.6	0.92
F-wave minimum latency	41.27 ± 6.41	40.13 ± 7.17	0.47
F-wave maximum latency	51.91 ± 2.97	51.96 ± 4.46	0.71
F-wave chronodispersion			
Normal	3 (15%)	7 (35%)	0.33
Delayed	6 (30%)	4 (20%)	
Absent	11 (55%)	9 (45%)	
Mean ± SD	12.64 ± 2.97	11.83 ± 3.29	0.25
**Posterior tibial nerve**			
Distal latency	4.93 ± 1.23	4.94 ± 1.04	0.99
Amplitude	13 ± 8.33	12.3 ± 5.25	0.65
NCV	44.07 ± 11.9	48.55 ± 5.75	0.10
F-wave mean latency			
Normal	11 (55%)	10 (50%)	0.53
Delayed	8 (40%)	10 (50%)	
Absent	1 (5%)	0 (0%)	
Mean ± SD	46.28 ± 4.34	47.98 ± 3.20	0.13
F-wave minimum latency	42.14 ± 6.7	45.86 ± 5.4	0.04*
F-wave maximum latency	50.35 ± 3.9	53.65 ± 4.29	0.01*
F-wave chronodispersion			
Normal	8 (40%)	7 (35%)	0.54
Delayed	11 (55%)	13 (65%)	
Absent	1 (5%)	0 (0%)	
Mean ± SD	9.36 ± 6.33	7.81 ± 4.07	0.23

NCV, nerve conduction velocity; SD, standard deviation.

a Significant *p*-value <0.05, paired *t*-test or Mc Nemar test, as appropriate.

The percentage of patients with a normal common peroneal nerve chronodispersion among cases increases in cases by 80%, while it increased by only 20% among controls. There is a highly statistical significant difference between the two groups (*p* < 0.01). Also, the percentage of patients with a normal posterior tibial nerve chronodispersion increased from 30 to 95% among cases, while it decreased from 40 to 35% in controls. There was a statistically significant difference between the two groups (*p* < 0.01).

## Discussion

The ultrasound-guided caudal epidural steroid injection is a well-known intervention for managing low back pain. It is a simple procedure that can be done in an outpatient clinic with no ionizing radiation or need for admission apart from short follow-up after the procedure. Its clinical efficacy is well-studied and proved by multiple studies. It shows much less risks and complications compared to the transforminal and interlaminar approaches. These advantages give the procedure a priority over other procedures while managing radiculopathic pain. However, its neurophysiologic impact has not been well investigated. In the current study, only the intervention group showed significant improvement of both the common peroneal and posterior tibial F-wave chronodispersion. Furthermore, the percentage of patients having an absent F wave for the common peroneal nerve (indicating severe root affection) reduced among cases from 45 to 15% post-intervention, while among controls it was reduced from 55% to only 45%.

The pathophysiology of nerve root injury is somewhat complex. Immunological, inflammatory, and neurochemical processes may all be critical, as may be ischemia.^24^ Due to their tight dural sleeve, the nerve roots are more prone to edema from compressive injury than peripheral nerves. In that sense, radiculopathies might best be viewed in the framework of entrapment neuropathies, as the roots become compressed by the fixed dura,^25^ leading to demyelination, and slowing of nerve conduction.^26^ For this reason, the F-wave parameters are useful in evaluating radiculopathies.

Most studies utilizing electrophysiological testing utilize needle electromyography pre-intervention to predict the outcomes of the injection.^27,28^ In the current study, it was found that the improvement of pain correlated with the improvement in electrophysiological parameters. It was also shown that patients with abnormalities in electrophysiological parameters pre-intervention had better pain and electrophysiological improvement.

Using F-waves for radiculopathy is not ideal as the injury may not affect the entire motor axons in the affected root. Aminoff et al^16^ (a cross-mark article in the use of F-waves for radiculopathy) evaluated 28 patients with radiculopathy (L5 and/or S1) showed a disappointing diagnostic yield of F-waves; only 5/28 patients. All of these had needle electromyographic abnormalities.

Recent studies used F-wave parameters as well as minimal F-wave latencies showed a sensitivity comparable to the use of needle electromyography regarding L5/S1 radiculopathy. A study of patients with L5/S1 radiculopathy using F-wave latencies and chronodispersion, needle EMG abnormalities were in 70% and F-wave abnormalities were found in 69%.^18^ F-wave abnormalities were detected in 13/23 patients where the only electromyographic denervation was in the paraspinal muscles, providing unique evidence for anterior rami injury. Another study on 95 patients with L5, S1, or L5 and S1 root lesions, F-waves were abnormal in 70% of the patients and needle electromyography in 77%. The gold-standard was surgery or myelography.^29^ The authors used chronodispersion and mean F-wave duration and concluded that the F-wave is clinically useful in evaluation of radiculopathy regardless to the needle electromyography or clinical findings. The improvement in F-wave parameters has been also correlated with recovery following surgery,^30^ providing evidences of the value of F-waves as follow-up study post-interventions as proved in the current study.

There are two main limitations to our study. First, there may have been an underestimation of the effect of ESIs on electrophysiological parameters, since some patients may have shown more improvement after multiple injections. For example, in a comprehensive review on ESI by MacVicar et al,^31^ the authors determined that 6% of individuals will require more than one injection to obtain a successful outcome. Second, the study did not evaluate the long-term effect of the tested interventions, since the outcome measures were only recorded once, 2 weeks after the intervention. Several studies were done evaluating the long-term efficacy of the image-guided spine injections with no focus on the electrophysiologic benefits.^32–34^ The relatively small sample size is also a limitation of this study.

In conclusion, the ultrasound-guided caudal epidural steroid injection was proved to be effective in terms of electrophysiologic short-term benefits for treatment of patients with radicular chronic low back pain.
